# Hierarchical Nanostructures of Iron Phthalocyanine Nanowires Coated on Nickel Foam as Catalysts for the Oxygen Evolution Reaction

**DOI:** 10.3390/molecules29174272

**Published:** 2024-09-09

**Authors:** Xianying Meng, Peng Yu, Mingyi Zhang

**Affiliations:** Key Laboratory for Photonic and Electronic Bandgap Materials, Ministry of Education, School of Physics and Electronic Engineering, Harbin Normal University, Harbin 150025, China

**Keywords:** electrocatalysis, phthalocyanine, OER, solvothermal

## Abstract

In this paper, iron phthalocyanine nanowires on a nickel foam (FePc@NF) composite catalyst were prepared by a facile solvothermal approach. The catalyst showed good electrochemical oxygen evolution performance. In 1.0 M KOH electrolyte, 289 mV low overpotential and 49.9 mV dec^−1^ Tafel slope were seen at a current density of 10 mA cm^−2^. The excellent electrochemical performance comes from the homogeneous dispersion of phthalocyanine nanostructures on the surface of the nickel foam, which avoids the common agglomeration problem of such catalysts and provides a large number of active sites for the OER reaction, thus improving the catalytic performance of the system.

## 1. Introduction

Water splitting for hydrogen production has an ideal development prospect as an energy production method [1,2]. As an important step in this process, the oxygen evolution reaction (OER) has attracted increasing attention. The four-electron transfer process and slow kinetics of OER have always hindered its industrial application [3,4,5]. Electrocatalysts are undoubtedly an important factor in improving OER performance. At present, the best materials used as OER catalysts are still limited to precious metal-based materials. Obviously, the high price limits the practical application of such catalysts. Therefore, how to develop efficient and inexpensive OER catalysts has become a research hotspot in this field [6,7,8,9,10,11].

Among the myriad of non-precious metal electrocatalysts, transition metal nitride–carbon compounds (M-Nx/C, where M = Fe, Co, Ni, Mo, etc.) have garnered significant scientific interest due to their exceptional performance in the oxygen reduction reaction (ORR) and oxygen evolution reaction (OER), coupled with their low cost, long lifespan, and environmental friendliness. Research has demonstrated that the catalytic activity of these materials has reached or even surpassed commercial Pt/C catalysts, positioning them as the most promising alternatives to platinum-based catalysts [12,13,14,15,16,17,18,19]. Theoretical calculations reveal that in these catalysts, the transfer of electrons from the active centers formed by nitrogen-containing groups coordinated with metal atoms to the O_2_ orbit weakens the O=O double bond, thereby facilitating the ORR process [20,21,22,23,24,25,26,27].

In the early stages of material development, M-Nx/C catalysts were typically synthesized via direct physical mixing and annealing of carbon–nitrogen and metal-containing precursors. However, due to the sluggish diffusion of metal atoms, this solid-state reaction inevitably led to a random distribution of C-N species in the final product, hindering the uniform distribution of metal atoms with C-N and subsequently reducing the generation of active sites [28,29,30]. Metal phthalocyanines and their derivatives emerge as ideal precursors for one-step synthesis of M-Nx/C. As macrocyclic compounds, metal phthalocyanines can encapsulate various elements such as iron, copper, cobalt, nickel, calcium, sodium, magnesium, and zinc within their rings. Notably, the unique C-N coordination structure with metals in these materials enables a molecular-level uniformity in the M-Nx/C structure [21,22,23,24,25,26,27,28,29,30,31,32,33,34,35,36].

In the thorough investigation presented in this paper, we harnessed a straightforward and efficient solvothermal method to securely anchor iron phthalocyanine (FePc) nanowires onto the porous and highly conductive nickel foam (NF) substrate. This innovative design not only retains the active sites of FePc as an effective catalyst but also significantly accelerates electron transfer at the catalytic interface through the superior conductivity of the nickel foam. In the critical oxygen evolution reaction (OER) tests, this composite catalyst demonstrated impressive performance, particularly in a 1.0 M KOH alkaline environment, where it achieved a low overpotential of 289 mV at a current density of 10 mA cm^−2^, significantly outperforming numerous similar catalysts. Remarkably, the catalyst also displays exceptional electrochemical durability, maintaining stable performance even after prolonged electrolysis, attributed primarily to its unique multi-level heterogeneous structural design. This architecture not only optimizes the surface activity of the catalyst but also enhances the efficient diffusion and separation of reactants and products, creating seamless electron pathways that further boost catalytic efficiency and stability. This work not only opens new avenues for the synthesis of phthalocyanine-based catalysts but also provides invaluable guidance and inspiration for the design and fabrication of other high-performance catalysts.

## 2. Result and Discussion

### 2.1. SEM and TEM Images, EDX Spectra

**Figure 1:**
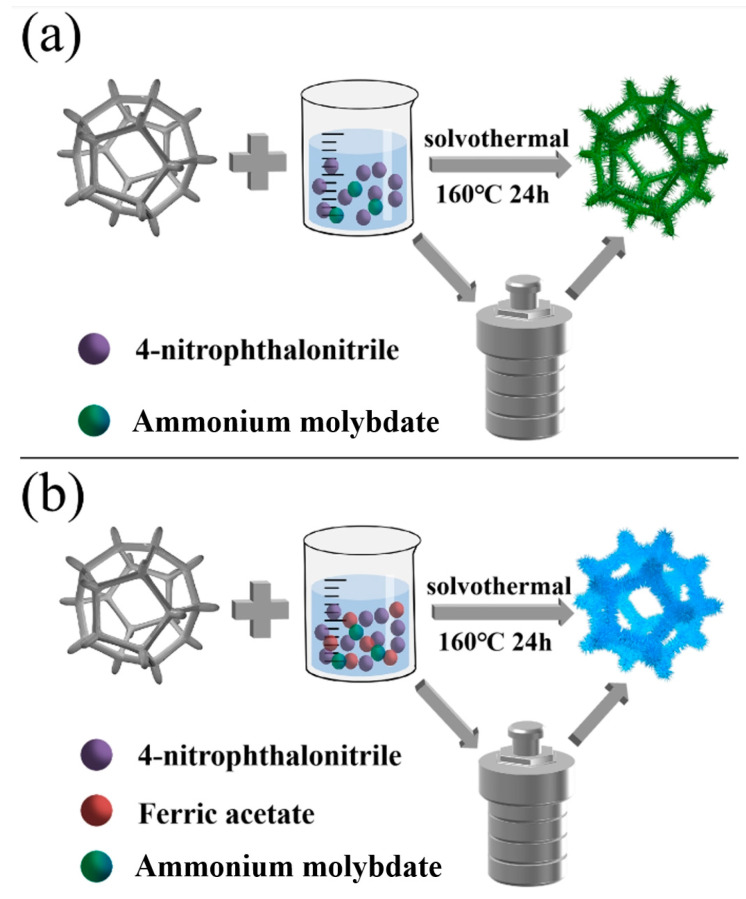
Schematic illustration of the fabrication of (**a**) NiPc@NF and (**b**) FePc@NF.

Figure 1 shows a schematic diagram of the synthesis process of FePc@NF and NiPc@NF. In the early stage of the formation of the phthalocyanine compound, 4-nitrophthalonitrile is relatively uniformly dispersed in the glycol solution, and then the phthalocyanine molecules are formed by chelating with the nickel atoms on the surface of the nickel foam. In the next stage of secondary growth, the subsequently generated NiPc will gather around the initially formed NiPc to grow and, finally, grow further into NiPc@NF. The synthesis mechanism of FePc@NF is similar to that of NiPc@NF. As shown in Figure 1b, during the synthesis of iron phthalocyanine, due to the addition of iron acetate during the synthesis process, the subsequently generated FePc grows in situ on the surface of the nickel foam and further prepares FePc with a uniform load. In the second process, the nitro group in phthalonitrile plays an important role in making the phthalocyanine molecules more tightly bonded to the surface of the nickel foam.

**Figure 2:**
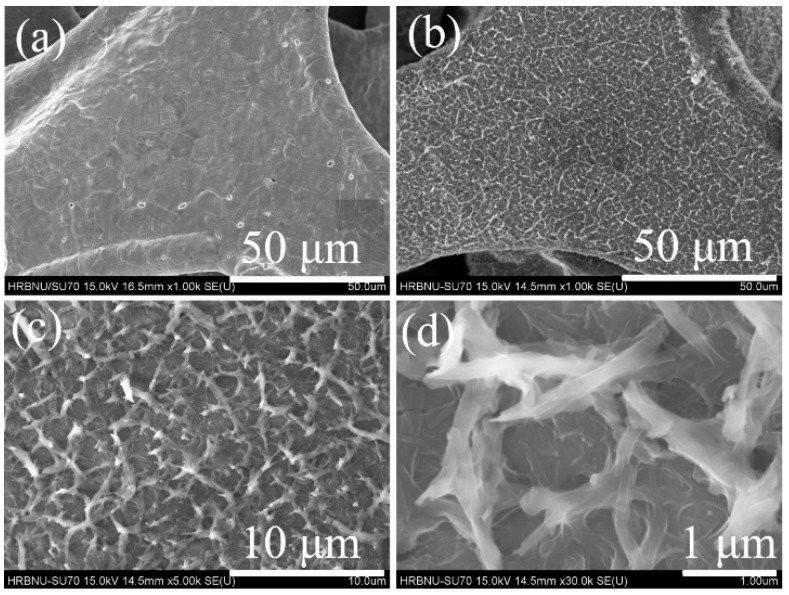
The SEM images of nickel foam (**a**), NiPc@NF (**b**–**d**).

To comprehensively unravel the microscopic morphological characteristics of the prepared samples, we conducted detailed characterizations using high-resolution scanning electron microscopy (SEM). Figure 2a displays the SEM image of pure nickel foam, clearly showcasing the meticulously treated nickel foam substrate with an extremely smooth surface, devoid of any secondary structures or minute protrusions. This indicates that the substrate surface achieved an ideal level of cleanliness and smoothness during the pretreatment stage. However, upon introducing phthalonitrile into the reaction system and subjecting it to a meticulously designed solvothermal reaction process, dramatic changes occur on the surface of the nickel foam. As depicted in Figure 2b–d, this series of progressively magnified SEM images vividly illustrates that the nickel foam surface is uniformly and densely covered with a layer of nanoscale wire-like structures. These nanowires resemble a finely woven net, tightly adhering to the skeleton of the nickel foam, forming a unique composite structure. This phenomenon provides compelling evidence that nickel phthalocyanine (NiPc) was not only successfully synthesized but also grown in a highly uniform manner on the surface of the nickel foam, demonstrating the precision and effectiveness of the synthesis strategy. Upon closer inspection, it can be observed that the diameters of these nanowires are approximately 200 nanometers, with uniform sizes and orderly arrangements, showcasing excellent morphological control. Nevertheless, it is noteworthy that the metal source in the reaction system primarily relies on the nickel atoms on the surface of the nickel foam, which to some extent limits the yield of NiPc. Consequently, while the nanowires cover a significant portion of the nickel foam surface, there are still uncovered blank areas.

**Figure 3:**
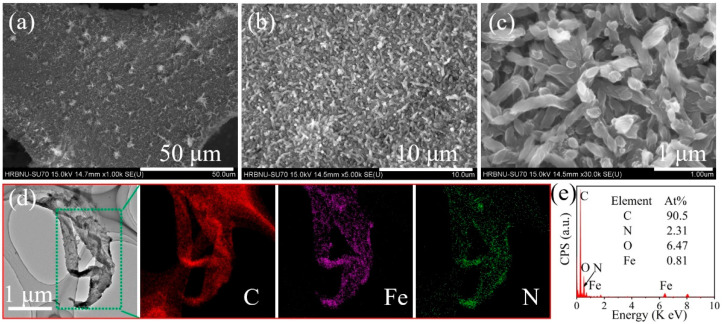
The SEM image of FePc@NF (**a**–**c**). The TEM image of FePc@NF and corresponding elemental mappings of C, Fe, and N of FePc@NF (**d**); the EDX spectra of FePc@NF (**e**).

In stark contrast to the NiPc, the surface morphology of the nickel foam underwent a remarkable transformation when iron ions were introduced into the reaction system as the new central ligand for phthalocyanine. As clearly depicted in Figure 3a–c, while the diameter of the iron phthalocyanine (FePc) nanowires remained relatively unchanged compared to the previously prepared NiPc nanowires, maintaining at approximately e 200 nanometers, a significant increase in density was observed. This notable change can be primarily attributed to the introduction of iron ions, which greatly accelerated the formation rate of phthalocyanine molecules in the ethylene glycol solution, resulting in a marked increase in the number of FePc nanowires loaded on the nickel foam surface. This substantial enhancement in active material provides the composite system with an abundance of catalytic sites, hinting at a potential leap in its catalytic performance. To further substantiate this conclusion, we conducted transmission electron microscopy (TEM) and corresponding energy-dispersive X-ray spectroscopy (EDX) analysis (Figure 3d,e). The results demonstrated that the prepared iron phthalocyanine-modified nickel foam (FePc@NF) composite material was primarily composed of elements such as C (90.5%), O (6.47%), N (18.87%), and Fe (0.81%), with the rational proportions of each element aligning with our expectations. Furthermore, the diameter of the iron phthalocyanine nanowires was consistent with the observations made by SEM. Notably, the EDX elemental mapping images offered a direct visualization of the distribution of these elements within the composite, showcasing their uniform and compact arrangement, collectively forming this unique nanostructure. The above results show that we successfully constructed iron phthalocyanine nanostructures on the surface of nickel foam by the solvothermal method.

### 2.2. Fourier Transform Infrared (FT-IR) Spectra

**Figure 4:**
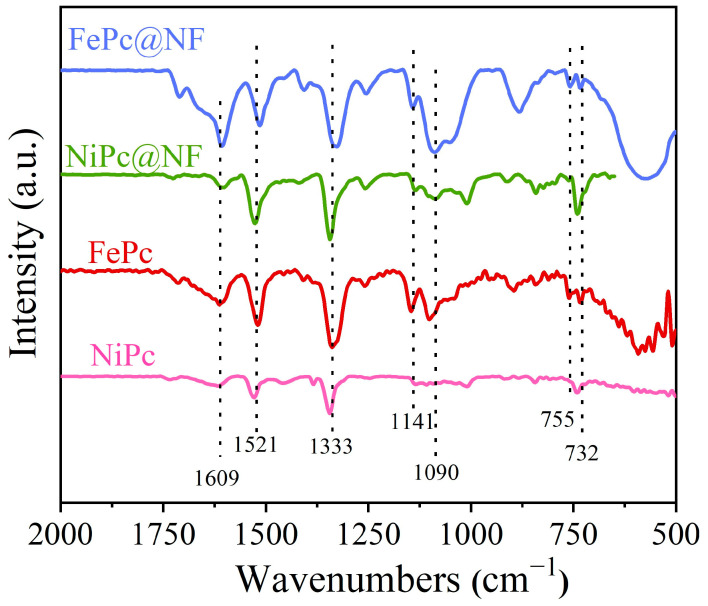
FT-IR spectra of the samples.

To further conclusively validate the presence of FePc, we meticulously employed Fourier-transform infrared (FT-IR) spectroscopy, an advanced technique, to conduct a comprehensive and insightful characterization of the carefully prepared material structures. In the spectral analysis presented in Figure 4, the samples of FePc@NF, pure FePc, and NiPc@NF all distinctly exhibit a series of characteristic vibration peaks, located approximately at 732, 755, 1090, 1141, and 1609 cm^−1^. These prominent peaks serve as direct evidence of the phthalocyanine skeleton and metal–ligand interactions, firmly demonstrating the existence and integrity of the FePc molecular structure. Furthermore, additional vibration peaks observed at 1521 and 1333 cm^−1^ in the spectra, upon careful comparison and analysis, are likely to represent the characteristic vibrations of the N-O bond within nitro groups in the molecular structure. This discovery aligns with descriptions found in the literature [37], further enriching our understanding of the chemical structure of the material. These exhaustive and precise FT-IR spectral analysis results not only conclusively prove the successful and stable existence of FePc molecules but also reveal their uniform distribution and secure attachment to the surface of the nickel foam.

### 2.3. X-ray Photoelectron Spectroscopy (XPS) Spectra

**Figure 5:**
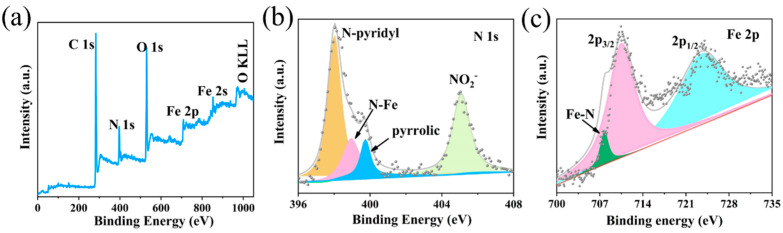
The survey XPS spectra of (**a**) FePc@NF. The high-resolution XPS spectra of (**b**) N1s and (**c**) Fe2p.

In order to study the surface composition and binding energy of the samples, FePc@NF samples were characterized by the XPS method. The full-scan spectrum in Figure 5a shows the presence of C, N, O, and Fe elements in the FePc nanowires. Figure 5b is the XPS spectrum of N1s, where the binding energy at 398.4 eV is attributed to pyridinyl N, the peak observed at 399.6 eV is due to the interaction of pyridinyl N with Fe, and the peak at 405.7 eV is due to NO^2−^. Figure 5c is an XPS spectrum of Fe _2p_, where the strongest pair of double peaks of Fe _2p3/2_ and Fe _2p1/2_ signals are seen at 711 eV and 725 eV, indicating the presence of metallic iron and iron oxide, with the peak at 706.9 eV attributed to the Fe-N bond [38]. All the above results also confirmed the formation of FePc molecules.

### 2.4. Oxygen Evolution Reaction (OER) Activity

**Figure 6:**
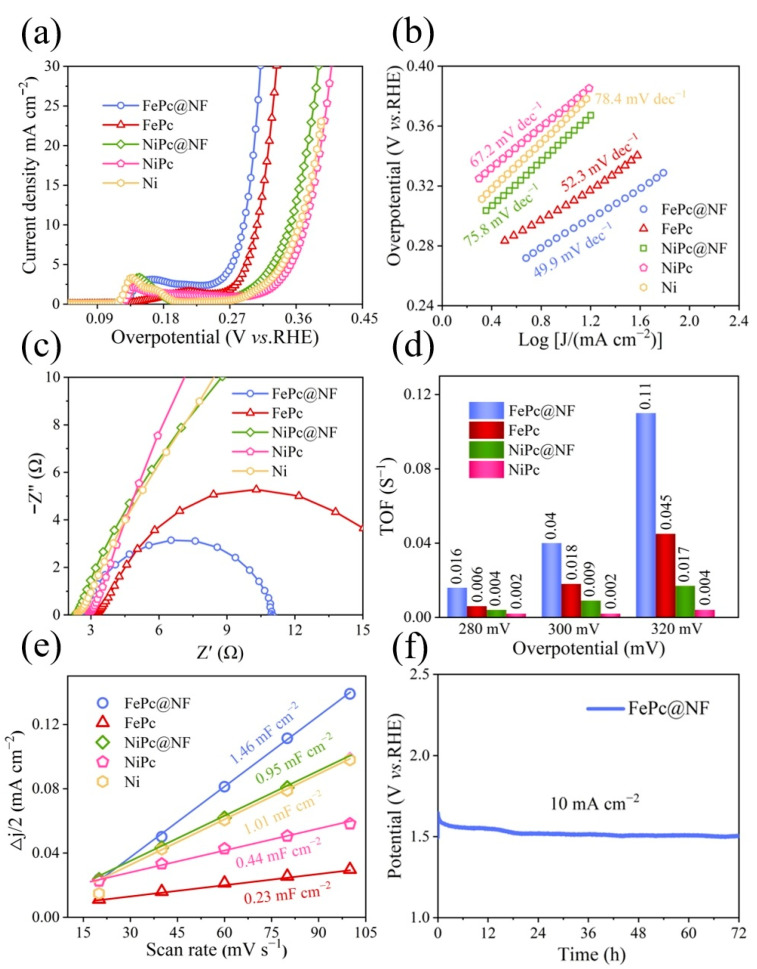
(**a**) LSV curves of the FePc@NF, FePc, NiPc@NF, NiPc and Ni. (**b**) Corresponding Tafel plots of the above catalysts. (**c**) EIS pattern of the above catalysts, investigated at a constant potential of 0.5V (vs. SCE). (**d**) TOF values of FePc@NF), FePc, NiPc@NF and NiPc. (**e**) △J/2 as a function of the scan rate of FePc@NF, FePc, NiPc@NF, NiPc, and Ni. (**f**) Long-term stability of FePc@NF at the current density of 10 mA cm^−2^ for 72 h.

In delving into the electrocatalytic performance of the oxygen evolution reaction (OER), we employed a three-electrode system with 1 M KOH as the electrolyte to comprehensively evaluate the performance of various materials. Figure 6a vividly displays the OER activity polarization curves of the electrocatalysts after 95% iR compensation. Notably, when the current density is set at 10 mA cm^−2^, FePc@NF exhibits an ultra-low overpotential of merely 289 mV, significantly outperforming its counterparts such as FePc (306 mV), NiPc@NF (352 mV), NiPc (372 mV), and pure Ni (366 mV), highlighting its immense potential in reducing energy consumption. Furthermore, Figure 6b illustrates the unique advantages of FePc@NF in the kinetic reaction process through Tafel slope analysis. With a slope value of 49.9 mV dec^−1^, it notably surpasses NiPc@NF (67.2 mV dec^−1^), NiPc (75.8 mV dec^−1^), and Ni (78.4 mV dec^−1^), indicating that FePc@NF is capable of facilitating electron transfer more efficiently, thereby accelerating the reaction process. To gain a more comprehensive understanding of the electrochemical behavior of FePc@NF, we utilized electrochemical impedance spectroscopy (EIS) technology. As depicted in Figure 6c, the charge transfer resistance (R_ct_) of FePc@NF is significantly lower than that of other comparative materials, reinforcing its exceptional ability to enhance electron transport efficiency and, consequently, ensure the efficient progression of the OER. Additionally, we further evaluated the electrocatalytic activity of the materials through the crucial indicator of turnover frequency (TOF). Figure 6d clearly illustrates the relationship between TOF and overpotential, revealing that regardless of overpotential variations, FePc@NF consistently maintains the highest active site density. This outstanding performance can be attributed to its unique nanostructure, which provides abundant channels for reaction intermediates to efficiently access the active centers, thereby promoting rapid reaction kinetics. To delve deeper into the electrochemical active surface area (ECSA) of FePc@NF, we conducted double-layer capacitance cyclic voltammetry (CV) tests. As shown in Figure 6e, the electrochemical double-layer capacitance (C_dl_) value of FePc@NF reaches as high as 1.46 mF cm^−2^, far exceeding those of FePc (0.23 mF cm^−2^), NiPc@NF (0.95 mF cm^−2^), NiPc (0.44 mF cm^−2^), and Ni (1.01 mF cm^−2^). As C_dl_ directly reflects ECSA, this result conclusively demonstrates that the FePc@NF electrode possesses more active sites during the OER, providing a robust foundation for efficient catalysis. Lastly, we rigorously tested the durability of FePc@NF. In practical applications, electrocatalysts must not only exhibit excellent catalytic performance but also maintain stable physical and chemical properties over extended periods. As illustrated in Figure 6f, a potentiostatic test (95% iR compensation) conducted in 1 M KOH solution at a current density of 10 mA cm cm^−2^ for 72 h reveals that the overpotential of FePc@NF remains virtually unchanged. This outcome conclusively validates the remarkable durability of the FePc@NF electrocatalyst under high current densities, laying a solid foundation for its widespread adoption in practical applications.

## 3. Experimental Section

### 3.1. Materials

4-Nitrophthalonitrile, ferric acetate, ammonium molybdate, and ethylene glycol were purchased from Zhiyuan Reagent (Tianjin, China). Analytical-grade chemicals that had not been further purified were used in this study.

### 3.2. Synthesis of Catalysts

In the synthesis process, we first weighed 4 mmol of 4-nitrophthalonitrile, 1 mmol of iron acetate, and 5.0 mg of ammonium molybdate as the catalyst. These were then combined in a solvent of 16 mL of ethylene glycol and thoroughly mixed through vigorous stirring to ensure uniform dispersion of all components. The resultant mixture was transferred into a 20 mL Teflon-lined stainless-steel autoclave, followed by the insertion of a pre-treated nickel foam substrate. The autoclave was immediately sealed. The entire reaction system was maintained at a temperature of 160 °C for 24 h to ensure complete progression of the chemical reactions. Upon completion of the reaction, we employed an ultrasonic-assisted method to thoroughly wash the reaction products three times with ethanol, aiming to remove any residual impurities and unreacted substances. Subsequently, the cleaned samples were placed in a constant temperature oven at 60 °C for 12 h of drying, resulting in the formation of iron phthalocyanine nanostructures uniformly loaded on the nickel foam (abbreviated as FePc@NF). To further delve into the dominant role of the NiFe composite system in catalytic performance and eliminate the interference from other potential byproducts, we devised a series of comparative experiments. One set of experiments employed the same synthesis method as above but omitted the addition of nickel foam, directly collecting and drying the generated precipitate to obtain unsupported iron phthalocyanine samples. These samples were used to evaluate the influence of the substrate material on catalytic performance. In another set of comparative experiments, we synthesized nickel foam-supported nickel phthalocyanine nanostructures (abbreviated as NiPc@NF). In this process, except for omitting iron acetate, all the other steps remained identical to the synthesis of FePc@NF. By collecting and drying the residual precipitate from the NiPc@NF solution, we also prepared nickel phthalocyanine samples without nickel foam support (abbreviated as NiPc).

### 3.3. Material Characterization

X-ray photoelectron spectroscopy (XPS, Thermo Fisher Scientific Company, Carlsbad, CA, USA), scanning electron microscopy (SEM; SU70, Hitachi, Tokyo, Japan), a transmission electron microscope (TEM, FEI, Hillsboro, OR, USA, Tecnai TF20), and a Fourier transform infrared spectrometer (FT-IR, Bruker, Ettlingen, Germany) were used to study the crystal structure and morphology of the material.

### 3.4. Electrochemical Measurements

In this study, electrochemical measurements of each electrode were performed on the VMP3 Electrochemical Workstation (Bio-Logic, Seyssinet-Pariset, France). In the classic three-electrode system, 1 M KOH (60 mL) was used as the electrolyte, a 1 cm × 1 cm platinum sheet electrode was employed as the counter electrode, and an Ag/AgCl electrode served as the reference electrode. The working electrode production method is as follows: FePc@NF and NiPc@NF are uniformly loaded on the nickel foam during the solvothermal process, which can be directly intercepted and tested, while FePc and NiPc need to make electrodes; the electrode size is 0.5 cm × 0.5 cm, and the load mass of the active substance is about 2.5 mg cm^−2^. Polyvinylidene fluoride is used as a binder. The electrochemical properties of the catalysts were characterized by linear sweep voltammetry, Tafel slope, electrochemical impedance spectroscopy, cyclic voltammetry (CV), and chronopotentiometry (CP) curve. Unless specified otherwise, the potential was converted to the reversible hydrogen electrode (RHE) scale using the equation: E_(RHE)_ = E_(SCE)_ + 0.241 + 0.059 pH. Electrochemical activation of these electrocatalysts was achieved through redox cycling. Linear sweep voltammetry (LSV) was performed at a scanning rate of 5 mV/s. Electrochemical impedance spectroscopy (EIS) was conducted across a frequency range from 100 kHz to 0.01 Hz, with an applied potential of 500 mV. The electrochemically active surface area (ECSA) value can be obtained from the following equation: ECSA = *C*_dl_/*C*s. *C*s is a general specific capacitance of 0.04 mF cm^−2^ in 1.0 M alkaline media for metal electrodes. The electrochemical double-layer capacitance (*C*_dl_) of the catalysts at different scanning rates was measured by CV. The turnover frequency (TOF) is calculated using the following equation:

$$\mathrm{TOF}\;\left(s^{-1}\right)=\frac{J\times N_{A}}{4\times F\times C},\;C=\frac{\int V\times A}{v\times 1.602\times 10^{-19}}$$

where *J* is the current density at 1.65 V_RHE_ (A cm^−2^), *N*_A_ is Avogadro’s number (6.023 × 10^23^), *F* is the Faraday constant (96,485 C mol^−1^), *C* is the surface density of atoms, and *ν* is the scan rate in CV curves (V s^−1^).

## 4. Conclusions

We innovatively employed the hydrothermal method to successfully synthesize a composite electrode material featuring uniform loading of iron phthalocyanine nanowires on nickel foam and thoroughly investigated its performance as a catalyst in the oxygen evolution reaction (OER). Experimental results indicate that this composite material exhibits an outstanding overpotential of just 289 mV at a current density of 10 mA cm^−2^. Remarkably, even after a continuous 72 h cycling test, its performance remained stable, demonstrating exceptional durability and stability. This superior electrochemical performance can be primarily attributed to the uniform and dense dispersion of the iron phthalocyanine nanostructures on the nickel foam substrate. This distribution pattern effectively mitigates the aggregation issue commonly encountered in traditional catalysts, thereby significantly increasing the number of active sites available for interaction with reactants. The abundance of active sites not only enhances electron transport efficiency but also facilitates the rapid formation and conversion of intermediates during the OER process, ultimately leading to a marked improvement in the catalytic performance of the system. In summary, our research findings offer novel insights and strategies for the development of highly efficient and stable OER catalysts.

## Data Availability

All the relevant data are included in this published article.

## References

[1] Yang D., Gu Y., Yu X., Lin Z., Xue H., Feng L. (2018). Nanostructured Ni_2_P-C as an Efficient Catalyst for Urea Electrooxidation. ChemElectroChem.

[2] Liu X., Zhang L., Li L., Ye X., Chen H., Wei Z. (2020). Mo_2_N–Ni/NF Heterostructure Boosts Electrocatalytic Hydrogen Evolution with Pt-Like Activity. Inorg. Chem..

[3] Chen J.G., Jones C.W., Linic S., Stamenkovic V.R. (2017). Best Practices in Pursuit of Topics in Heterogeneous Electrocatalysis. ACS Catal..

[4] Chandrakala K.B., Giddaerappa, Reddy K.R.V., Shivaprasad K.H. (2022). Investigational undertaking descriptors for reduced graphene oxide-phthalocyanine composite based catalyst for electrochemical oxygen evolution reaction. J. Electroanal. Chem..

[5] Kumar Y., Kozlova E.J., Kikas A., Kaarik M., Aruvali J., Kisand V., Leis J., Tamm A., Tammeveski K. (2021). Bimetal Phthalocyanine-Modified Carbon Nanotube-Based Bifunctional Catalysts for Zinc-Air Batteries. ChemElectroChem.

[6] Murthy A.P., Theerthagiri J., Madhavan J. (2018). Insights on Tafel Constant in The Analysis of Hydrogen Evolution Reaction. J. Phys. Chem. C.

[7] Sun H.M., Xu X.B., Yan Z.H., Chen X., Cheng F.Y., Weiss P.S., Chen J. (2017). Porous Multishelled Ni_2_P Hollow Microspheres as Active Electrocatalyst for Hydrogen and Oxygen Evolution. Chem. Mater..

[8] Dai L.M., Xue Y.H., Qu L.T., Choi H.J., Beak J.B. (2015). Metal-Free Catalysts for Oxygen Reduction Reaction. Chem. Rev..

[9] Ziani A., Shinagawa T., Stegenburga L., Takanabe K. (2016). Generation of Transparent Oxygen Evolution Electrode Consisting of Regularly Ordered Nanoparticles from Self-Assembly Cobalt Phthalocyanine as a Template. ACS Appl. Mater. Interfaces.

[10] Helsel N., Choudhury P. (2023). Investigation of bifunctionality of FePc-functionalized graphene for enhanced ORR/OER activity. Mol. Catal..

[11] Li J.W., Liu P., Mao J.X., Yan J.Y., Song W.B. (2021). Two-dimensional conductive metal-organic frameworks with dual metal sites toward the electrochemical oxygen evolution reaction. J. Mater. Chem. A.

[12] Lo Vecchio C., Arico A.S., Monforte G., Baglio V. (2018). EDTA-derived Co-N-C and Fe-N-C Electro-Catalysts for The Oxygen Reduction Reaction in Acid Environment. Renew. Energy.

[13] Wu G., More K.L., Johnston C.M., Zelenay P. (2011). High-Performance Electrocatalysts for Oxygen Reduction Derived from Polyaniline. Iron Cobalt Sci..

[14] Arul A., Pak H., Moon K.U., Christy M., Oh M.Y., Nahm K.S. (2018). Metallomacrocyclic carbon complex: A study of bifunctional electrocatalytic activity for oxygen reduction and oxygen evolution reactions and their lithium-oxygen battery applications. Appl. Catal. B—Environ..

[15] Isvoranu C., Wang B., Ataman E., Schulte K., Knudsen J., Andersen J.N., Bocquet M.L., Schnadt J. (2011). Ammonia adsorption on iron phthalocyanine on Au (111): Influence on adsorbate-substrate coupling and molecular spin. J. Chem. Phys..

[16] Matsuda S., Mori S., Hashimoto K., Nakanishi S. (2014). Transition Metal Complexes with Macrocyclic Ligands Serve as Efficient Electrocatalysts for Aprotic Oxygen Evolution on Li_2_O_2_. J. Phys. Chem. C.

[17] Kumar Y., Kibena-Poldsepp E., Kozlova J., Rahn M., Treshchalov A., Kikas A., Kisand V., Aruvali J., Tamm A., Douglin J.C. (2021). Bifunctional Oxygen Electrocatalysis on Mixed Metal Phthalocyanine-Modified Carbon Nanotubes Prepared via Pyrolysis. ACS Appl. Mater. Interfaces.

[18] Abbaspour A., Mirahmadi E. (2013). Electrocatalytic activity of iron and nickel phthalocyanines supported on multi-walled carbon nanotubes towards oxygen evolution reaction. Electrochim. Acta.

[19] Kumar A., Zhang G., Liu W., Sun X. (2022). Electrocatalysis and activity descriptors with metal phthalocyanines for energy conversion reactions. J. Electroanal. Chem..

[20] Shen M.X., Zheng L.R., He W.H., Ruan C.P., Jiang C.H., Ai K.L., Lu L.H. (2015). High-Performance Oxygen Reduction Electrocatalysts Derived from Uniform Cobalt-Adenine Assemblies. Nano Energy.

[21] Yamada Y., Kura J., Toyoda Y., Tanaka K. (2021). High catalytic methane oxidation activity of monocationic μ-nitrido-bridged iron phthalocyanine dimer with sixteen methyl groups. Dalton Trans..

[22] Zhu Y.S., Zhang B.S., Liu X., Wang D.W., Su D.S. (2014). Unravelling The Structure of Electrocatalytically Active Fe-N Complexes in Carbon for The Oxygen Reduction Reaction. Angew. Chem.-Int. Ed..

[23] Liu Z.L., Liu C.Y., Bian L.Z., Qi J., Yang L.L., Wei P.Y., Fu P., Han S.W., Han W., Hu Z.X. (2024). *In-situ* construction of Ni–Fe alloy nanoparticles on perovskite surface for CO_2_ direct electrolysis. Int. J. Hydrogen Energy.

[24] Kuznetsova I., Lebedeva O., Kultin D., Mashkin M., Kalmykov K., Kustov L. (2024). Enhancing efficiency of nitrate reduction to ammonia by Fe and Co nanoparticle-based bimetallic electrocatalyst. Int. J. Mol. Sci..

[25] Du Y.F., Su X., Wang X., Ye L.T., Xie K. (2024). In situ exsolved CoFe alloys over perovskite toward enhanced ammonia synthesis. New J. Chem..

[26] Abdelghafar F., Xu X.M., Guan D.Q., Lin Z.Z., Hu Z.W., Ni M., Huang H.T., Bhatelia T., Jiang S.P., Shao Z.P. (2024). New nanocomposites derived from cation nonstoichiometric Ba*_x_*(Co, Fe, Zr, Y)O_3-*δ*_ as efficient electrocatalysts for water oxidation in alkaline solution. ACS Mater. Lett..

[27] Xu X.M., Zhong Y.J., Wajrak M., Bhatelia T., Jiang S.P., Shao Z.P. (2024). Grain boundary engineering: An emerging pathway toward efficient electrocatalysis. InfoMat.

[28] Xu X.M., Shao Z.P., Jiang S.P. (2022). High-entropy materials for water electrolysis. Energy Technol..

[29] Yuceel C., Sahin Z., Isci U. (2022). Substituent effect on iron phthalocyanines as cyclohexene oxidation catalysts. J. Porphyr. Phthalocyanines.

[30] Jia H.X., Yao Y.C., Zhao J.T., Gao Y.Y., Luo Z.L., Du P.W. (2018). A novel two-dimensional nickel phthalocyanine-based metal-organic framework for highly efficient water oxidation catalysis. J. Mater. Chem. A.

[31] Shimizu T., Wakamatsu K., Yamada Y., Toyoda Y., Akine S., Yoza K., Yoshikawa H. (2021). Application of μ-Nitrido- and μ-Carbido-Bridged Iron Phthalocyanine Dimers as Cathode-Active Materials for Rechargeable Batteries. ACS Appl. Mater. Interfaces.

[32] Mihara N., Yamada Y., Takaya H., Kitagawa Y., Aoyama S., Igawa K., Tomooka K., Tanaka K. (2017). Oxygen Reduction to Water by a Cofacial Dimer of Iron (III)-Porphyrin and Iron (III)- Phthalocyanine Linked through a Highly Flexible Fourfold Rotaxane. Chem.-Eur. J..

[33] Yamada Y., Miwa Y., Toyoda Y., Phung Q.M., Oyama K.I., Tanaka K. (2023). Evaluation of CH_4_ oxidation activity of high-valent iron-oxo species of a μ-nitrido-bridged heterodimer of iron porphycene and iron phthalocyanine. Catal. Sci. Technol..

[34] Isvoranu C., Knudsen J., Ataman E., Schulte K., Wang B., Bocquet M.L., Andersen J.N., Schnadt J. (2011). Adsorption of ammonia on multilayer iron phthalocyanine. J. Chem. Phys..

[35] Yamada Y., Sugiura T., Morita K., Ariga-Miwa H., Tanaka K. (2019). Improved synthesis of monocationic μ-nitrido-bridged iron phthalocyanine dimer with no peripheral substituents. Inorg. Chim. Acta.

[36] Bata P., Notheisz F., Kluson P., Zsigmond A. (2015). Iron phthalocyanine as new efficient catalyst for catalytic transfer hydrogenation of simple aldehydes and ketones. Appl. Organomet. Chem..

[37] Guo Z., Mu J., Chen B. (2015). 3D Flower-Like Ironphthalocyanine Hierarchical Microstructures: Solvothermal-Fabrication and High Visible Light Photocatalytic Properties. Ceram. Int..

[38] Guo Z., Chen B., Mu J. (2012). Iron Phthalocyanine/TiO_2_ Nanofiber Heterostructures with Enhanced Visible Photocatalytic Activity Assisted with H_2_O_2_. J. Hazard. Mater..

